# Immunodeficiency and autoimmunity: companions not opposites

**DOI:** 10.1172/JCI162170

**Published:** 2022-08-15

**Authors:** David A. Fox

**Affiliations:** Division of Rheumatology, Department of Internal Medicine and Clinical Autoimmunity Center of Excellence, University of Michigan, Ann Arbor, Michigan, USA.

## Abstract

Autoimmunity has long been regarded as the polar opposite of immunodeficiency, but clinical and experimental evidence refute this notion. Indeed, numerous inborn or acquired immunodeficiency syndromes are characterized by the development of autoimmune complications in the setting of deficient immune defenses against microbial pathogens. Appreciation that much of the daily business of a healthy immune system is the avoidance of potentially harmful responses to innocuous environmental antigens or components of the host organism helps provide a context for these observations. In this issue of the *JCI*, Abt and colleagues report on purine nucleoside phosphorylase (PNP) deficiency, exploring the basis for the autoimmune complications that develop in this particular form of T cell immune deficiency and assigning a key role for overactivation of TLR7.

## Autoimmune diseases in the context of immune deficiencies

Purine nucleoside phosphorylase (PNP) mediates phosphorolysis of inosine, deoxyinosine, guanosine, and deoxyguanosine. In patients with PNP deficiency, guanosine, deoxyguanosine, and dGTP accumulate (1). Hypoplasia of the thymus and altered T cell selection occur, likely due to toxic levels of these purine metabolites. A wide variety of bacterial and viral infections can ensue, including disseminated Epstein-Barr virus and cytomegalovirus infections (1–3), with poor prognosis. Other organ systems are also affected. In the brain, a neuronal apoptosis pathway that is magnified in the absence of PNP may explain neurological symptoms (1–4). Allogeneic hematopoietic stem cell transplant has been reported to at least partly alleviate the neurological aspects of PNP deficiency while reconstituting T cell immunity (1–3). Protein replacement strategies and gene therapy are also being explored (1, 5).

Clear evidence for the frequent occurrence of autoimmune diseases in the context of primary immune deficiencies has been established in over 20 distinct immune deficiency syndromes (6). It is, therefore, hardly surprising that autoimmune disease has been reported in up to one-third of PNP-deficient patients, including occasional cases of systemic lupus (7). In the various primary immune deficiency conditions, multiple mechanisms may explain the occurrence of autoimmunity, including defects in thymic selection, inadequate numbers and/or function of regulatory T cells, and excessive antigen load due to tissue damage and exposure of cryptic self-antigens (6). These various immune malfunctions may arise from molecular defects in receptor signaling, costimulation, antigen presentation in the thymus or peripherally, complement function, antigen clearance, or apoptosis (6). Interestingly, depletion of complement, defects in antigen clearance, and either excessive or defective apoptosis can all be mechanistic features of flares of systemic lupus erythematosus.

## A role for TLR7 in human disease

In this issue of the *JCI*, a report by Abt and colleagues explored the consequences of deficiency of the enzyme purine nucleoside phosphorylase (PNP) (8). The authors used synthetic inhibitors of PNP to clarify the pathway through which PNP deficiency is toxic to developing T cells and also propose a mechanism for the association of PNP deficiency with autoimmunity through effects on TLR7. PNP inhibition enhanced the activation of TLR7 by increasing levels of its typical RNA ligands, leading to increased production of IL-6, especially by B lymphocytes. Increased formation of germinal centers also occurred, even in the absence of immunization with exogenous antigen. In MRL-LPR mice, which are prone to developing autoimmunity, acceleration of lymphoproliferative and lupus-like autoimmunity was observed with five weeks of exposure to a PNP inhibitor (8). Further work is needed to more fully understand how the accumulation of purine nucleosides alters the consequences of TLR7 engagement, the full range of cell types in which these interactions occur, and which cytokines, other than IL-6, play roles in the acceleration of autoimmunity by PNP inhibitors. Confirmation of inhibitor specificity will also be important to exclude confounding of the results by off-target effects on other receptors.

It is becoming increasingly clear that activation of TLR7, an innate immune receptor that is highly expressed by dendritic cells, B lymphocytes, and monocytes, is important in human autoimmune diseases. In rheumatoid arthritis, synovial macrophages release GU-rich microRNA ligands for TLR7, which induce TNF production via autocrine or paracrine effects (9). In mice, ligands of TLR7, acting through pathways that also require production of IFN- λ, can induce a model of systemic lupus (10). In a similar model the gut microbiome plays an essential role in the induction of lupus by stimulating TLR7. Notably, this model can be controlled by diet (11). Moreover, a recent report describes the severe presentation of systemic lupus in a seven-year-old girl with a gain-of-function *TLR7* variant who lacked other lupus-predisposing gene alleles (12), placing TLR7 among the growing list of single-gene causes for lupus, which is typically a polygenic condition. In this patient, the TLR7 gain of function not only heightened guanosine sensing, but also prolonged B cell survival (12). Moreover, the discovery of individuals with partial PNP deficiency (13) raises the possibility that subtle polymorphisms in *PNP* could also contribute to the pathogenesis of human autoimmune disease.

## Conclusions and implications

The plethora of signals to which the immune system responds creates numerous opportunities for inadequate response to infection and excessive response to self. The evolving understanding of PNP deficiency includes dual consequences of immune dysfunction that encompass both immune deficiency and autoimmunity. PNP deficiency is yet another example by which a single gene defect can create a diverse set of consequences that lead to immune imbalance; it also illustrates how metabolic derangements within lymphocytes can have powerful concurrent and opposing consequences. Moreover, understanding of the effects of subtle *PNP* variants could reveal unsuspected and widespread influences of PNP on human immune-mediated diseases.

## Version 1 08/15/2022

Electronic publication
